# In vivo label-free optical signatures of chemotherapy response in human pancreatic ductal adenocarcinoma patient-derived xenografts

**DOI:** 10.1038/s42003-023-05368-y

**Published:** 2023-09-25

**Authors:** Jaena Park, Janet E. Sorrells, Eric J. Chaney, Amro M. Abdelrahman, Jennifer A. Yonkus, Jennifer L. Leiting, Heidi Nelson, Jonathan J. Harrington, Edita Aksamitiene, Marina Marjanovic, Peter D. Groves, Colleen Bushell, Mark J. Truty, Stephen A. Boppart

**Affiliations:** 1 Beckman Institute for Advanced Science and Technology, University of Illinois Urbana-Champaign, Urbana, IL 61801 USA; 2 Department of Bioengineering, University of Illinois Urbana-Champaign, Urbana, IL 61801 USA; 3 Division of Hepatobiliary and Pancreas Surgery, Mayo Clinic, Rochester, MN 55905 USA; 4 Division of Research and Optimal Patient Care, Cancer Programs, American College of Surgeons, Rochester, MN 55905 USA; 5 Center for Individualized Medicine, Mayo Clinic, Rochester, MN 55905 USA; 6 NIH/NIBIB Center for Label-free Imaging and Multiscale Biophotonics, University of Illinois Urbana-Champaign, Urbana, IL 61801 USA; 7 National Center for Supercomputing Applications, University of Illinois Urbana-Champaign, Urbana, IL 61801 USA; 8 Department of Electrical and Computer Engineering, University of Illinois Urbana-Champaign, Urbana, IL 61801 USA; 9 Cancer Center at Illinois, University of Illinois Urbana-Champaign, Urbana, IL 61801 USA; 10 Interdisciplinary Health Sciences Institute, University of Illinois Urbana-Champaign, Urbana, IL 61801 USA

**Keywords:** Chemotherapy, Multiphoton microscopy, Cancer microenvironment, Cancer metabolism, Predictive markers

## Abstract

Pancreatic cancer is a devastating disease often detected at later stages, necessitating swift and effective chemotherapy treatment. However, chemoresistance is common and its mechanisms are poorly understood. Here, label-free multi-modal nonlinear optical microscopy was applied to study microstructural and functional features of pancreatic tumors in vivo to monitor inter- and intra-tumor heterogeneity and treatment response. Patient-derived xenografts with human pancreatic ductal adenocarcinoma were implanted into mice and characterized over five weeks of intraperitoneal chemotherapy (FIRINOX or Gem/NabP) with known responsiveness/resistance. Resistant and responsive tumors exhibited a similar initial metabolic response, but by week 5 the resistant tumor deviated significantly from the responsive tumor, indicating that a representative response may take up to five weeks to appear. This biphasic metabolic response in a chemoresistant tumor reveals the possibility of intra-tumor spatiotemporal heterogeneity of drug responsiveness. These results, though limited by small sample size, suggest the possibility for further work characterizing chemoresistance mechanisms using nonlinear optical microscopy.

## Abstract (web summary)

Label-free multi-modal nonlinear optical microscopy is used to investigate distinct structural and functional features in patientderived xenografts tumor models in mice under chemotherapy treatment.

## Introduction

Pancreatic cancer is a deadly disease with a five-year survival rate of only 11%^1^. The most prevalent type of pancreatic cancer is pancreatic ductal adenocarcinoma (PDAC). Elusive symptoms of PDAC often result in late-stage diagnosis when the tumor is already inoperable^2^. Many patients who initially respond to anti-cancer monotherapy such as gemcitabine treatment develop resistance to the treatment within a few weeks^3, 4^. Therefore, after PDAC diagnosis, a more effective combination therapy is used. The standard-of-care chemotherapy includes folinic acid, 5-fluorouracil (5-FU), irinotecan (Irino), and oxaliplatin (OX) (combined as FOLFIRINOX) or gemcitabine and nab-paclitaxel (combined as Gem/NabP). However, despite the diverse mechanisms of action of these anti-cancer agents, most PDAC tumors are not fully eradicated with chemotherapy treatment, primarily due to the high prevalence of chemoresistance^5^. The biomolecular mechanisms of intrinsic or acquired tumor resistance to combined chemotherapy regimens are not well understood, although it is suspected that the significant alterations in energy metabolism and intercellular communication pathways might be involved^2,3,6,7^.

Extremely high intra- and inter-tumoral heterogeneity, such as the presence of more than one clone of cancer cells within a given tumor mass and the presence of different genetic alterations in different metastatic lesions from a single patient, is one of the primary reasons why a metastatic PDAC tumor is challenging to treat, and even more challenging to predict the efficacy of that treatment^2,6,8,9^. PDAC tumors can exhibit KRAS, TP53, SMAD4, and CDKN2A gene mutations, which activate intracellular signaling pathways, triggering aggressive phenotypic and metabolic changes within tumor cells^10^. These metabolic alterations are intertwined with the structural characteristics of the tumor microenvironment. Dense extracellular matrix (ECM), primarily consisting of collagen, results in poor oxygen perfusion of tumor tissue. This dense ECM, among other factors, promotes a severely hypoxic microenvironment^6^. This hypoxic microenvironment is implicated in cellular reprogramming of energy metabolism, which can result in increased glucose and glutamine absorption, lactate dehydrogenase (LDH) expression^11^, and chemoresistance^12^. While LDH-A inhibitors have been shown to improve gemcitabine sensitivity in PDAC cell lines, implicating LDH-A as a biomarker of resistance^13^, in general, there is a lack of specific and sensitive preclinical prognostic markers indicative of tumor responsiveness or resistance to treatment^14^.

One additional element of the tumor microenvironment recently gaining interest is extracellular vesicles (EVs). EVs are sub-micron bilipid membrane-bound particles released by all cell types that play a role in cell-to-cell communication^15,16^. One recent study implicated EVs in the PDAC microenvironment as mediators of cellular adaptation to hypoxia- and chemotherapy-related changes^17^. Spatial mapping of the metabolic heterogeneity, ECM, and EVs within the PDAC tumor microenvironment can provide a better overall understanding and shed light on the relationship of these structural and functional characteristics to chemoresistance over the course of treatment.

Translational in vivo models of cancer progression are essential for studying the complexities of the tumor microenvironment and changes in chemotherapy response over time. Standard histological staining of sectioned tumors provides detailed two-dimensional information about anatomical structures and cellular constituents of these tissues, but lacks functional and metabolic information and requires significant preparation time. Standard histopathology can be complemented by methods such as real-time in vivo optical imaging. To relate studies directly to patient outcomes and to create a pathway for personalized medicine approaches, characterization of individual patient PDAC tumors is needed. To meet these requirements, patient-derived xenografts (PDX) can be implanted into mice to capture the tumor heterogeneity found in human subjects^18,19^, which is not available in cell culture models. PDX models allow for the investigation of nuances associated with systemic chemotherapy treatment and allow for in vivo characterization. The response of PDX tumors to treatment has shown a strong correlation with patient response to treatment in various conditions^20^, including PDAC response to chemotherapy^21^. PDX mouse models of tumors have been characterized using a variety of imaging methods, including in vivo fluorescent-label-based imaging systems, X-ray computed tomography, positron emission tomography, and magnetic resonance imaging. However, these imaging methods all have limited spatial resolution that do not allow for cellular and subcellular examination, including cell-cell heterogeneity and small features such as EVs.

A multi-modal, high-resolution optical imaging system is required to image the intact in vivo PDAC microenvironment and provide insight on the structural and functional heterogeneity. Additionally, to circumvent logistical issues with the administration of multiple fluorescent probes (exogenous or genetically-expressed) that potentially perturb the fragile native tumor microenvironment, label-free optical imaging methods are ideal for in vivo studies. Nonlinear optical methods allow for multiplexing several types of endogenous contrast and for better imaging penetration depth into in vivo tissues than single-photon fluorescence. Using precisely tailored ultrashort laser excitation pulses, simultaneous label-free autofluorescence multiharmonic (SLAM) microscopy can simultaneously excite multiple label-free nonlinear optical contrasts within biological samples to provide a high-resolution ( < 500 nm) structural and metabolic map of the tissue^22^. As shown previously, four endogenous nonlinear optical imaging modalities are used in SLAM microscopy: second (SHG) and third (THG) harmonic generation, and two-photon (2PF) and three-photon (3PF) excited autofluorescence imaging. The SHG channel detects collagen; the THG channel detects heterogeneity in refractive indices, often boundaries between cells and within the tissue; the 2PF channel detects flavin adenine dinucleotide (FAD) autofluorescence; and the 3PF channel detects reduced nicotinamide adenine dinucleotide and reduced nicotinamide adenine dinucleotide phosphate (NAD(P)H) autofluorescence^22^. FAD and NAD(P)H are key metabolic cofactors that provide insight on energy metabolism; both are involved in the electron transport chain and many other key metabolic pathways. For example, NADH is a cofactor for LDH and NADPH is a cofactor for NADPH oxidase enzymes. In addition to examining each channel individually, various other metrics can be examined, such as collagen alignment^23,24^, extracellular vesicle properties^25–27^, and the optical redox ratio (ORR). The ORR is calculated as the intensity of the 2PF channel over the sum of the 2PF and 3PF channels, to approximate the amount of FAD in proportion to the amount of FAD and NAD(P)H in order to assess mitochondrial metabolism of cells^22,28^. FAD, NAD(P)H, and ORR analysis have all been used to examine hypoxia and altered metabolism in different types of cancer^29–31^.

**Fig. 1:**
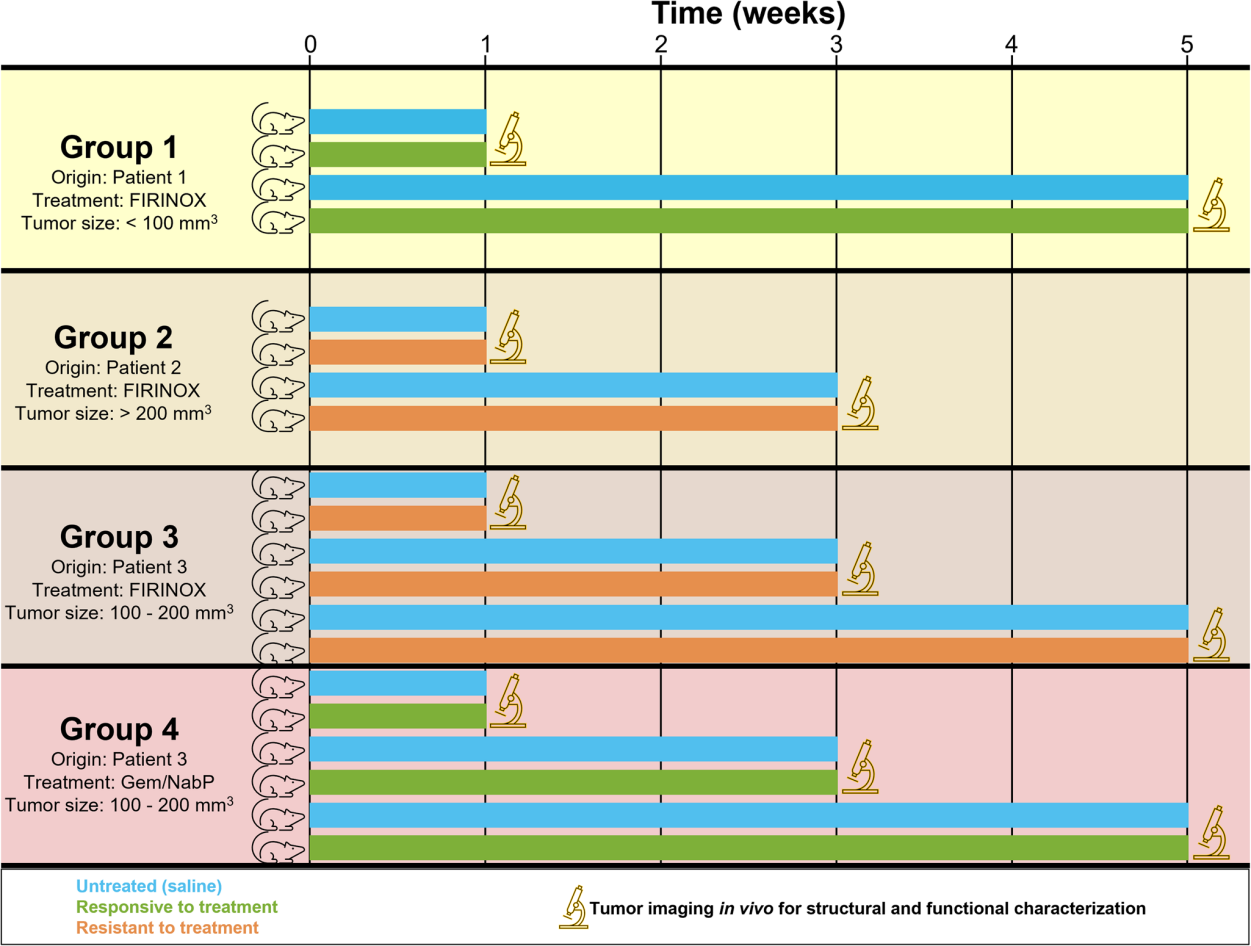
Schematic of study design to examine the feasibility of SLAM microscopy to observe and characterize PDAC tumors in PDX mice treated with two different chemotherapy regimens to which it is responsive or resistant. Four different groups were examined with various combinations of tumor origin, chemotherapy treatment, and starting tumor size. Once tumors reached the starting size, chemotherapy regimens were administered until the predetermined timepoint for in vivo imaging. A total of 20 mice were used; one mouse was examined for each combination of timepoint (1 week, 3 weeks, or 5 weeks), treatment (saline injections for untreated control or chemotherapy treatment), and group (1, 2, 3, or 4).

The goal of this study was to investigate the capabilities of SLAM microscopy to determine the responsiveness and/or resistance of PDAC tumors in PDX mice throughout the course of treatment with chemotherapeutic agents. Due to its multi-modal approach and high-resolution metabolic characterization capabilities, SLAM microscopy can detect subtle and heterogenous responses. In this feasibility study, 20 tumor-bearing mice received intraperitoneal chemotherapy based on the standard of care chemotherapy for patients diagnosed with PDAC (FIRINOX, Gem/NabP, or saline injections for tumor-bearing untreated controls) over five weeks and tumors were imaged in vivo with SLAM microscopy at predetermined timepoints. PDX tumors originated from three different human patients and were treated with either FIRINOX or Gem/NabP; two experimental groups examined tumors responsive to treatment, and two experimental groups examined tumors resistant to treatment (Fig. 1). Groups 1 and 2 used PDX samples from different patients and examined the response to FIRINOX, and groups 3 and 4 used PDX samples from the same patient and examined the response to FIRINOX (group 3) and to Gem/NabP (group 4). Quantitative results of the optically-resolved tumor morphology and metabolism showed distinct evidence of responsiveness and resistance and indicate that all chemotherapy treatments elicited a similar initial metabolic surge over weeks 1 and 3, which continued until week 5 for the responsive tumor, but by week 5 returned to levels similar to the untreated control.

## Results

### Visualization and quantification of the in vivo PDX tumor microenvironment with SLAM microscopy

**Fig. 2:**
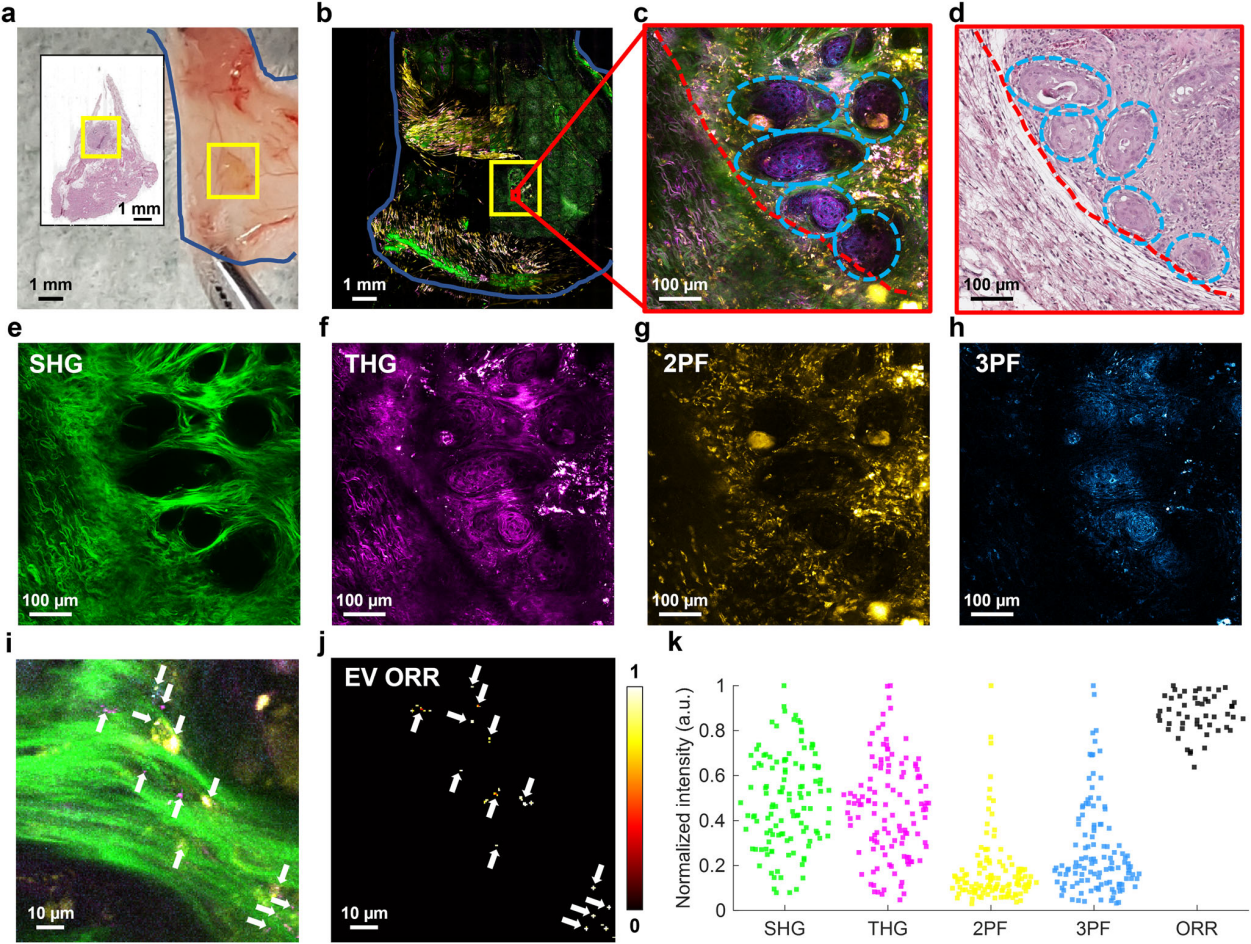
Qualitative and quantitative in vivo imaging of PDAC tumor in PDX mouse using SLAM microscopy. Example data is from a tumor responsive to FIRINOX (group 1) after 1 week of treatment. **a** Skin flap outlined in blue and approximate tumor in the yellow box. Inset is an H&E-stained histology image of the corresponding skin flap. **b** Large-area mosaic SLAM image of the skin flap in (**a**) with approximate tumor area located in yellow box. **c** Example FOV within the tumor from the red box in (**b**). The tumor margin is indicated with a red dashed line. The light blue dashed circles show clusters of cancer cells. **d** H&E-stained histology image of (**c**). **e** SHG, (**f**) THG, (**g**) 2PF, and (**h**) 3PF individual SLAM channels from (**c**). **i** Zoomed in the area from the central region of (**c**), with visible EVs indicated by small bright spots in the THG channel and annotated with white arrows. **j** EV segmentation mask, which enables quantification of EV density, EV ORR, and a fraction of NAD(P)H-rich EVs. **k** Example quantification of image channels and ORR from (**c**), with each point representing a 100×100 μm^2^ region within the tumor images.

At each predetermined timepoint, in vivo tumor imaging was performed with SLAM microscopy for quantitative and qualitative imaging (Fig. 2). The resultant SLAM images show a similar physical structure as fresh tissue and histology (Fig. 2a–d), and furthermore provides the basis for structural and functional tumor visualization and analysis that is not available in histology images (Fig. 2e–k). Based on previous work, EVs can be visualized as small dots with high THG signal (Fig. 2i) and are segmented (Fig. 2j) using a blob detection algorithm^32^. From these segmented EVs, the EV spatial density (in EVs/mm^2^), EV mean ORR, and a fraction of NAD(P)H-rich EVs can be estimated. Thus, to fully utilize the capabilities of SLAM microscopy, ten variables were examined to evaluate the structural and functional changes of the tumor microenvironment in response to chemotherapy treatment: SHG intensity, THG intensity, FAD intensity, NAD(P)H intensity, collagen alignment ratio, ORR, fraction of NAD(P)H-rich pixels (calculated as fraction of pixels with below-mean ORR), EV density, ORR of EVs, and fraction of NAD(P)H-rich EVs pixels (calculated as fraction of EVs with below-mean ORR. Interpretation and biochemical significance of image metrics in relation to cancer imaging is given in Table 1.

**Table 1:** Interpretation of image analysis metrics.

Image metric	Metric implications	Demonstrated relevance in cancer imaging
SHG intensity	SHG intensity increases with collagen content and can indicate changes in the collagen fiber internal structure.	• SHG of collagen in ex vivo human breast cancer shows differences between tumor bulk and tumor-stroma interfaces and differences before and after neoadjuvant chemotherapy^47^. • PDAC shows significantly more SHG from collagen than normal pancreatic cancer^48^.
THG intensity	THG intensity increases with optical heterogeneity within tissues and can be associated with features such as increased cellularity and EVs.	• THG images showed optical heterogeneity and increased cellularity in ex vivo human brain tumors^35^. • THG showed increased cellularity in the tumor microenvironment and immune cell recruitment in ex vivo rat mammary tumors^22,27^.
2PF (FAD) intensity, 3PF (NAD(P)H) intensity, and ORR	FAD intensity increases with electron transport chain activity. NAD(P)H intensity increases with glycolysis and decreases with electron transport chain activity. FAD and NAD(P)H intensity and lifetime are often used in calculating a variety of image analysis metrics such as ORR and optical metabolic index (OMI). ORR increase indicates a relative increase in mitochondrial metabolism.	• NAD(P)H fluorescence intensity and lifetime increase as human breast cancer cell lines undergo apoptosis^33, 34^. • Decreased ORR and increased metabolic activity were shown in precancerous epithelia of in vivo hamster oral cancer^30^. • OMI showed changes in human and mouse PDAC tumor organoids based on the type of organoid and its treatment^31^.
Collagen alignment ratio	Collagen remodeling often occurs in cancer, resulting in some tumors with more aligned collagen.	• A graph neural network was used to differentiate pancreatic cancer and chronic pancreatitis based on coregistered histology and SHG of collagen for ex vivo human samples^49^. • High alignment in collagen is associated with worse survival in ex vivo human PDAC samples^50^. • More aligned collagen is associated with better response to therapy in ex vivo breast cancer^47^. • Collagen in the canine ex vivo mammary tumor has a higher alignment ratio than in the tumor margin^23^.
Fraction of NAD(P)H-rich pixels and a fraction of NAD(P)H-rich EVs	Higher NAD(P)H levels may be indicative of more glycolytic metabolism in cells, which is also shown in EVs.	• There was an increased fraction of NAD(P)H-rich EVs in in vivo and rat mammary tumor tissues^25,26^ and ex vivo human breast tumor tissues^25^. • The fraction of NAD(P)H-rich EVs in dog-urine-derived EVs in cases of bladder cancer showed an increase compared to non-cancerous control^51^.
EV density	Increased number of extracellular vesicles is often associated with tumors and metastatic potential.	• Increased EV density was observed in in vivo rat mammary tumor tissues^25–27^ and ex vivo human breast tumor tissues^25^.
EV ORR	Decreased EV ORR may indicate changes in signaling related to parent cell metabolism in tumors.	• Decreased EV ORR in in vivo rat mammary tumor tissues and ex vivo human breast tumor tissues was observed^25^.

Image metrics used for analysis in this study are given individually or grouped together based on similar characteristics in the left column, and the general implications of each metric/group of metrics is given in the middle column. The right column gives select examples of each metric/group of metrics being used in previous studies using nonlinear optical imaging for cancer diagnosis and/or response to chemotherapy treatment.

SLAM microscopy can be used for both in vivo and ex vivo imaging. One tumor specimen with a matched FOV was imaged in vivo and ex vivo on the same day after the mouse was sacrificed and the tumor was excised. This was achieved via careful manipulation, handling, and positioning of the tumor throughout excision and imaging. While some metrics (collagen alignment ratio, fraction of NAD(P)H-rich pixels, EV mean ORR, and fraction of NAD(P)H-rich EVs) did not show significant differences between in vivo and ex vivo, the mean intensity of all four channels showed significantly increased intensity, the tissue ORR was significantly decreased, and the EV density was significantly increased in the ex vivo tissue (Fig. S1).

### Structural and functional analysis to assess PDX tumor responsiveness to chemotherapy treatment

**Fig. 3:**
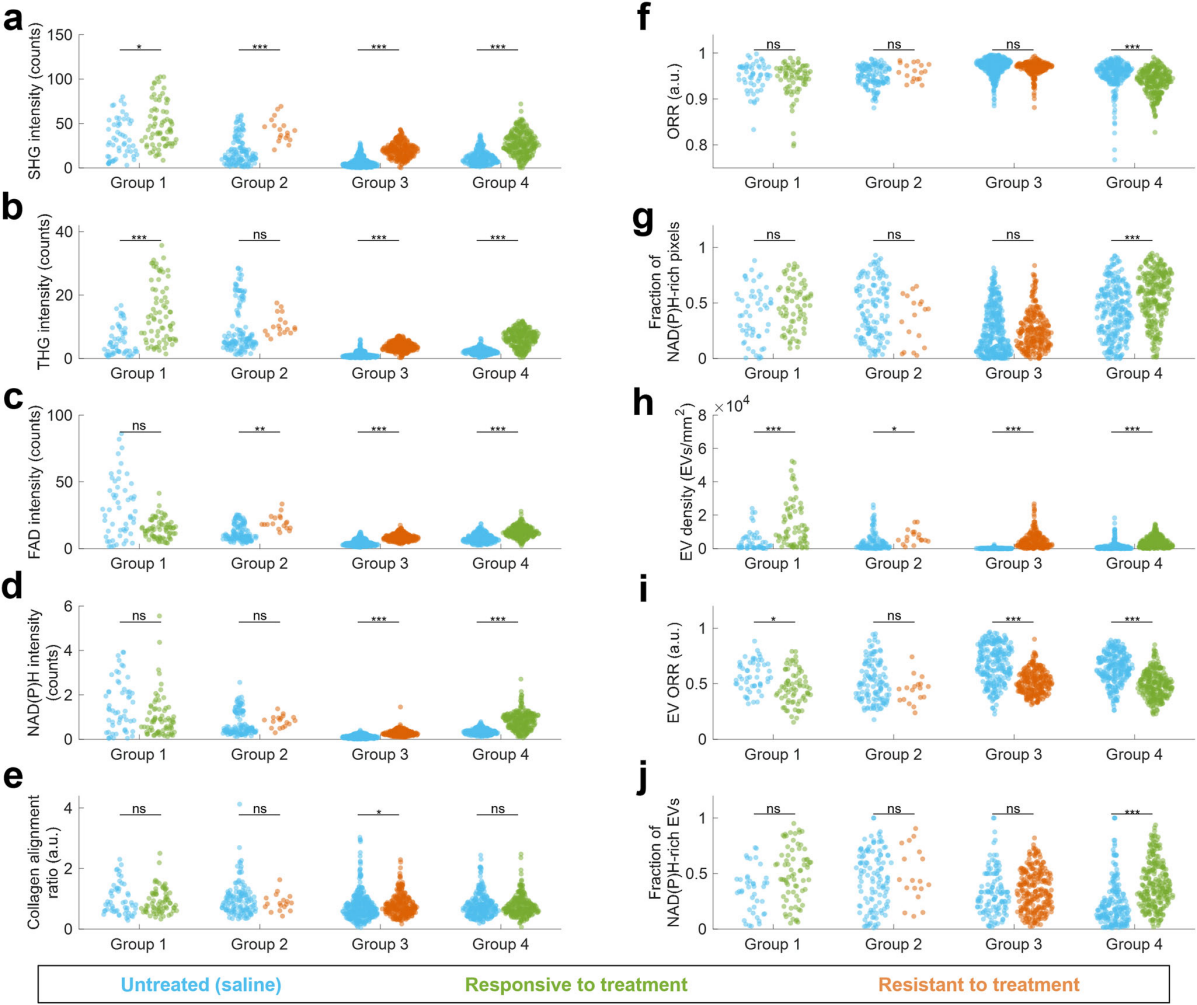
Structural and functional analysis of PDAC tumor response to chemotherapy treatment for all groups. All mosaic images were divided into tiles of 600×600 pixels (300×300 μm^2^), all timepoints were pooled together, and the following image metrics were computed for each tile: **a** SHG mean intensity in photon counts per pixel, **b** THG mean intensity in photon counts per pixel, **c** 2PF FAD mean intensity in photon counts per pixel, **d** 3PF NAD(P)H mean intensity in photon counts per pixel, **e** collagen alignment ratio computer from Fourier analysis of SHG channel, **f** mean optical redox ratio (ORR) of segmented tumor region within tile, **g** fraction of NAD(P)H-rich pixels of segmented tumor region within tile, defined as pixels with ORR < 0.8, **h** segmented EV density in EVs/mm^2^, **i** mean EV ORR, **j** fraction of NAD(P)H-rich EVs, defined as the fraction of EVs within the tile with ORR < 0.8. For groups 1 and 2, *n* = 2 mice; for groups 3 and 4, *n* = 3 mice. ns: not significant; *: *p* < 0.05; **p < 0.01; ***p < 0.001.

To evaluate the overall effect of the treatment, mice from all weeks were pooled together for each group and all ten structural and functional metrics were examined (Fig. 3). Groups 1 and 2 both showed no significant differences between timepoints across all metrics, groups 3 and 4 both showed significant differences between timepoints in a few metrics as discussed in the following section. All groups showed significant increases in SHG intensity and EV density in treated groups compared to untreated control. Groups 1 and 4, both responsive to treatment, showed significant differences in four and nine of the ten metrics, respectively. Responsive groups both showed increased SHG, THG, EV density, and EV ORR, and no change in collagen alignment ratio. Groups 2 and 3, both resistant to treatment, showed significant differences in three and seven metrics, respectively. Both resistant groups exhibited increased SHG, FAD, and EV density, and no change in ORR and fraction of NAD(P)H-rich pixels or EVs. Except for ORR and EV ORR, all significant differences between treated and untreated controls showed an increase in the metric for the treated group, regardless of whether the tumor was responsive or resistant to treatment. Groups 1, 2, and 3 were all treated with FIRINOX, and all show similar increases in SHG intensity and EV density, without a significant difference in ORR, fraction of NAD(P)H-rich pixels, and fraction of NAD(P)H-rich EVs.

Furthermore, to examine the role of inter- and intra-group variability, significant differences between untreated control groups were examined by pooling data for all timepoints (Fig. S2). Results indicated that no image metric was consistent for all untreated control mice, but the collagen alignment and EV ORR were not significantly different for mice implanted with tumor tissues from the same patient. However, a different number of fields of view were acquired for each sample based on the visible tumor area, leading to differences in the relative proportion of data collected at each timepoint for this comparison.

### Tumor resistant to chemotherapy shows an initial reaction similar to tumor responsive treatment but reverses over time

**Fig. 4:**
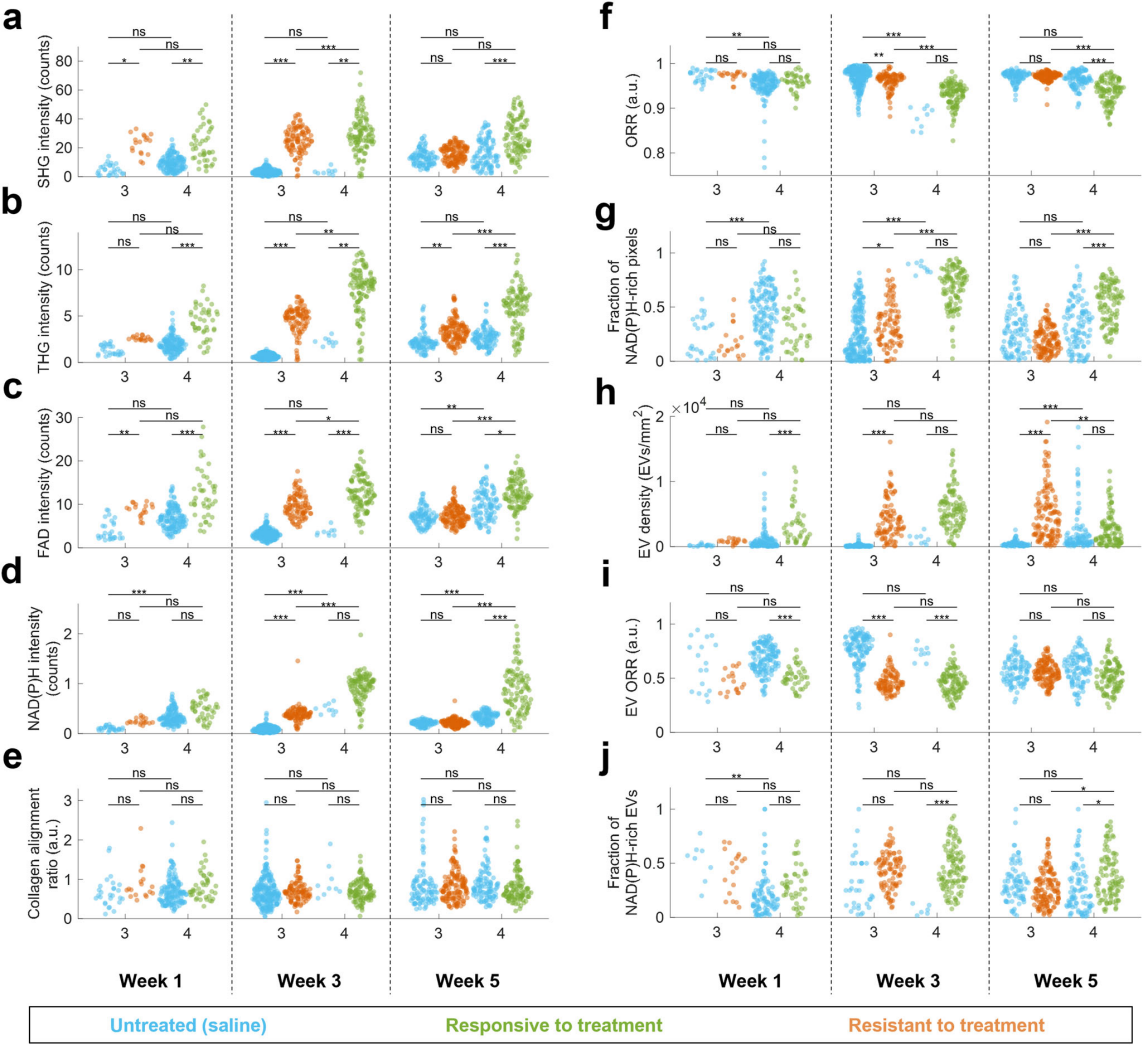
Structural and functional analysis of PDAC tumor response to chemotherapy treatment for responsive (group 4) and resistant (group 3) over five weeks of treatment. All mosaic images were divided into tiles of 600×600 pixels (300×300 μm^2^) and the following image metrics were computed for each tile: **a** SHG mean intensity in photon counts per pixel, **b** THG mean intensity in photon counts per pixel, **c** 2PF FAD mean intensity in photon counts per pixel, **d** 3PF NAD(P)H mean intensity in photon counts per pixel, **e** collagen alignment ratio computer from Fourier analysis of SHG channel, **f** mean optical redox ratio (ORR) of segmented tumor region within tile, **g** fraction of NAD(P)H-rich pixels of segmented tumor region within tile, defined as pixels with ORR < 0.8, **h** segmented EV density in EVs/mm^2^, **i** mean EV ORR, **j** fraction of NAD(P)H-rich EVs, defined as the fraction of EVs within the tile with ORR < 0.8. For each timepoint, group, and treatment, *n* = 1 mouse. ns: not significant; *: *p* < 0.05; **p < 0.01; ***p < 0.001.

The changes in features over weeks 1, 3, and 5 were examined in groups 3 and 4, which used tumor tissues originating from the same patient (Fig. 4). It is important to note that for each group, one mouse was imaged per treatment per timepoint, which means interanimal variability is a confounding variable. To determine which metrics would be robust to this interanimal variability, the untreated control animals (with the same PDX tumors but receiving saline injections instead of chemotherapy) from groups 3 and 4 were compared. These animals were not litter mates and received saline injections at different frequencies, but both represent untreated tumor tissues from the same patient. In addition to collagen alignment ratio and EV ORR, which showed no significant difference for the untreated controls from groups 3 and 4 with all weeks pooled together (Fig. S2), both SHG intensity and THG intensity showed no significant differences for the two untreated control groups across all timepoints, indicating that these are the most robust metrics when interanimal variability is present. Additionally, FAD intensity, EV density, and fraction of NAD(P)H-rich EVs showed no significant difference between untreated control groups for two of the three timepoints and should be considered somewhat robust but not completely reliable when examining data with interanimal variability. For variables where the two untreated control groups showed significant differences, it is not possible to untangle the interanimal variability from the effect of the chemotherapy with complete certainty within this dataset.

When comparing the treated resistant and responsive tumors, similar trends are observed across weeks 1 and 3 and across many metrics (Fig. 4, Table 2): both groups showed elevated optical heterogeneity via increased THG and some collagen remodeling via increased SHG. The resistant tumor showed elevated energy metabolism via significantly (*p* < 0.05) increased FAD, NAD(P)H, and fraction of NAD(P)H-rich pixels and decreased ORR, whereas the responsive tumor appears to follow the same trends but only showed a statistically significant increase in FAD intensity. In terms of EVs, both groups showed elevated EV density, and altered EV composition via decreased EV ORR and/or increased fraction of NAD(P)H-rich EVs compared to untreated controls. For the responsive tumor (group 4), all of these trends continue to week 5 with the exception of EV density, which decreased to a value similar to the untreated control. However, by week 5, the resistant tumor (group 3) showed a response much less similar to the responsive tumor and more similar to the untreated control. Metrics such as SHG, FAD, NAD(P)H, collagen alignment ratio, ORR, fraction of NAD(P)H-rich pixels, EV ORR, and a fraction of NAD(P)H-rich EVs settled to levels not significantly different from the untreated control; only THG and EV density remained significantly elevated.

**Table 2:** Summary of resistant and responsive tumor metrics over time.

	Early response (week 1 and/or week 3)	Indicative response (week 5)
**Resistant**	High heterogeneity and cell debris: • THG intensity increased (ns/***)	High heterogeneity and cell debris: • THG intensity increased (**)
	Elevated energy metabolism: • FAD intensity increased (**/***) • NAD(P)H intensity increased (ns/***) • ORR decreased (ns/**) • Fraction of NAD(P)H-rich pixels increased (ns/*)	Decreased energy metabolism / consistent with untreated control: • FAD intensity no change (ns) • NAD(P)H intensity no change (ns) • ORR no change (ns) • Fraction of NAD(P)H-rich pixels no change (ns)
	Elevated number of EVs with altered cargo: • EV density increased (ns/***) • EV ORR decreased (ns/***) • Fraction of NAD(P)H-rich EVs increased (ns/ns)	Elevated number of EVs with cargo consistent with untreated control: • EV density increased (***) • EV ORR no change (ns) • Fraction of NAD(P)H-rich EVs no change (ns)
	Some collagen remodeling: • SHG intensity increased (*/***) • Collagen alignment ratio increased (ns/ns)	No collagen remodeling: • SHG intensity no change (ns) • Collagen alignment ratio no change (ns)
**Responsive**	High heterogeneity and cell debris: • THG intensity increased (***/**)	High heterogeneity and cell debris: • THG intensity increased (***)
	Slightly elevated energy metabolism: • FAD intensity increased (***/***) • NAD(P)H intensity no change (ns/ns) • ORR no change (ns/ns) • Fraction of NAD(P)H-rich pixels no change (ns/ns)	Elevated energy metabolism: • FAD intensity increased (*) • NAD(P)H intensity increased (***) • ORR decreased (***) • Fraction of NAD(P)H-rich pixels increased (***)
	Elevated number of EVs with altered cargo: • EV density increased (***/ns) • EV ORR decreased (***/***) • Fraction of NAD(P)H-rich EVs increased (ns/***)	Number of EVs consistent with untreated control with altered cargo: • EV density not significantly different (ns) • EV ORR no change (ns) • Fraction of NAD(P)H-rich EVs increased (*)
	Some collagen remodeling: • SHG intensity increased (**/**) • Collagen alignment ratio no change (ns/ns)	Some collagen remodeling: • SHG intensity increased (***) • Collagen alignment ratio no change (ns)

The early response is generalized based on the response seen in weeks 1 and 3 of treatment, where responsive and resistant tumors showed similar trends. The left column gives the response at week 5, which is indicative of whether the tumor is responsive or resistant to treatment. Significance compared to matched untreated control is indicated in parenthesis.

## Discussion

Due to the high inter- and intra-tumor heterogeneity in PDAC, there is a critical need for methods to evaluate the in vivo PDAC microenvironment with sub-cellular resolution and with capabilities to characterize both tumor morphology and metabolism. SLAM microscopy has been used to provide structural and functional characterization in a variety of cancer types^23,25–27^, but until this study SLAM microscopy had not been applied for in vivo PDAC imaging. To enable this, tumors from PDAC patients with different responses to chemotherapy were implanted into PDX mice, which could be readily accessed by surgical opening of a skin flap. Label-free, high-resolution, live imaging of the PDAC microenvironment using SLAM microscopy provides unique insight into the biochemical context surrounding the role of metabolism and extracellular vesicles in differences between drug responsiveness and resistance. From SLAM images, ten image metrics were extracted and compared to analyze the effect that responsive and resistant chemotherapy has on structural and function microenvironmental features.

Both drug regimens used in this study inhibit DNA synthesis and repair, mechanisms that are not directly visible with SLAM microscopy. However, SLAM microscopy provides quantitative and interpretable metabolic information on many other cellular mechanisms related to the chemotherapeutic response, such as ATP production, glycolytic vs. mitochondrial metabolism, and cell death. Group 4, the only group treated with Gem/NabP, was the only group to show an overall decrease in ORR and overall increases in the fraction of NAD(P)H-rich pixels and EVs (Fig. 3), which could possibly indicate that Gem/NabP is affecting the relative ratio of mitochondrial vs. glycolytic metabolism more than FIRINOX. Elevated FAD levels are seen in group 4, as well as in the resistant groups 2 and 3, and increased NAD(P)H levels are seen in groups 3 and 4. These increased intensities may be due to increases in overall cellular energy metabolism. Increased energy metabolism and ATP production can be due to drug-induced cellular apoptosis^33,34^, but are also associated with a variety of interrelated mechanisms such as the drug efflux pump P-glycoprotein (P-gp) that is related to with multidrug resistance^11^. NAD(P)H autofluorescence lifetime has also been used to characterize signals from both apoptosis and necrosis^34^, and could be considered for use in future studies. Groups 1, 3, and 4 showed increased THG signal in treated tumors (Fig. 3). Increased THG signal may be due to an increase in cell debris from apoptosis or increased tumor cellularity^22,26,35^. No metrics showed clear trends to discriminate responsive from resistant tumors, however, this could be due to the heterogeneity of metabolic phenotype in PDAC^36^, which is visible in the differences between untreated control tumors (Fig. S2), the relatively small sample size used in this feasibility study, and the possibility of subpopulations of tumors cells that are sensitive to a treatment that is classified as resistant based on overall response.

Groups 3 and 4 provided comparable tumors that originated from the same patient with a known responsiveness to Gem/NabP and resistance to FIRINOX. Both treatments exhibited an initial response (weeks 1 and 3) consisting of high THG signals due to heterogeneity and cell debris, elevated FAD and NAD(P)H due to increased energy metabolism and possibly due to cell death, increased EVs with altered cargo, and slight collagen remodeling with increased SHG (Fig. 4, Table 2). At week 5, all variables remained altered for group 4, showing a consistent reaction to the chemotherapy known to be responsive. However, group 3, known to be resistant, showed a return in almost all metrics towards the untreated control values. The THG, FAD, NAD(P)H, ORR, and fraction of NAD(P)H-rich pixels all returned to values consistent with the untreated control tumor, signifying less cell death than in the previous weeks, whereas the responsive Gem/NabP-treated tumor maintained elevated THG, FAD, NAD(P)H, and fraction of NAD(P)H-rich pixels, and lowered ORR, signifying continued cell death and elevated metabolism (Table 2). At week 5, EV density remained high in the group 3 tumor resistant to treatment but the NAD(P)H and FAD levels in EVs returned to values consistent with the untreated control tumor, whereas in the responsive to treatment, the EV density fell to values consistent with the untreated control tumor but NAD(P)H- and FAD-related EV cargo remained significantly different than the untreated control (Fig. 4). These results suggest that the main metabolic characteristics of a resistant tumor may take up to five weeks to present, and that at least for these models of PDAC tumors, an initial metabolic surge may occur regardless of treatment responsiveness. PDAC tumors are extremely heterogenous, and likely some subpopulation of cells in the group 3 PDX tumors were responsive to the FIRINOX treatment, but by week 5 resistance had been acquired by those cells and/or other cells resistant to FIRINOX had expanded to become the dominant population within the tumor. These results correspond well with the fact that PDAC patients often initially respond to treatment, yet patients with PDAC have poor overall survival due to acquired resistance^3,37^. Furthermore, this biphasic response may support the beneficial role of chemotherapy switch in PDAC treatment to overcome subpopulations of cells that are resistant to one chemotherapy regimen and responsive to another^38^.

Elevated EV density was observed in both responsive (group 4) and resistant (group 3) tumors during the early response and a continued elevated EV density was observed at week 5 in the resistant tumor (Fig. 4). EVs have been implicated for their role in chemoresistance, and it is possible that the elevated EVs could confer resistance between cells^17^. For example, it has been shown in vitro that EVs can mediate the transfer of P-gp from drug-resistant cells to drug-sensitive cells^39,40^. Along with P-gp, many different proteins and nucleic acids have been identified in EVs as potential mediators of cancer drug resistance^41^. Additionally, as cells undergo apoptosis they release apoptotic bodies, so a subpopulation of these EVs may be resulting from cell death. Label-free imaging of EVs in the tumor microenvironment is a relatively new concept, and only a few studies have been performed to date^25–27^, none of which have studied the effect of drug treatment on EVs in vivo. The ability for SLAM microscopy to study EVs label-free in the in vivo tumor microenvironment thus provides a unique advantage over systems lacking THG, which is used for segmentation. EVs in this study were segmented in in vivo images based on previous methods^25,27,32^, but segmentation in this study was not validated on tagged EVs, which could help improve specificity for future work. Despite this, in vivo characterization of EVs in the tumor microenvironment remains a promising avenue for future work. Metabolic and EV-related information from SLAM microscopy provides a unique context for the functional aspect of the tissue, which is notably absent for H&E-stained histology images of the same tumors (Figs. S3, 4). EV analysis provides a new and promising avenue for label-free characterization of the live tumor microenvironment.

While initial results show strong evidence for the feasibility to study PDAC tumors in PDX mice using SLAM microscopy, this study was limited in ways that can be addressed in future studies. Inter-animal variability is present in these results since only one mouse was used for each group, treatment, and timepoint. This interanimal variability cannot be definitively untangled from the results due to the small sample size. The results of this study should be considered preliminary, and require replication at a larger scale to improve certainty. Different starting sizes of tumors were used to diversify the characteristics of the tumors examined, which showed that SLAM microscopy can assess tumors of different sizes but creates a barrier for clear quantitative comparison between groups. Future studies should examine a larger number of mice for each group and timepoint to account for the variability between animals and could use a starting tumor size anywhere within the range of those examined here. On the other hand, one barrier to future studies is that PDX mice models require human tissue, time, money, and energy to maintain and grow, which may hinder the feasibility of some large-scale studies. Patient-derived organoids have additionally been used for similar studies examining drug response^31,42^, although organoids not contained in an in vivo system, and instead are often grown under atmospheric oxygen levels in various growth matrices, so mimicking systemic administration of chemotherapy over time may present a challenge. Imaging in vivo provides specific benefits, such as the ability to image dynamic processes and observe blood vessel formation within samples. Furthermore, while the use of PDX mice serve as an incredibly useful approach for investigating the in vivo PDAC microenvironment, it is important to consider that this model cannot perfectly recapitulate the effect of chemotherapy applied to a human patient with PDAC. For example, the tumors are not located in the pancreas of the mouse, and the mouse size, relative tumor volume, source of nutrients, and lifespan are very different from those in humans. Another confounding factor is that the human patients were treated with neoadjuvant chemotherapy prior to tumor excision the implantation into mice, and the role that this neoadjuvant chemotherapy could have on the PDX tumors is not yet understood. Groups 1 and 4 used the same chemotherapy regimen that was given to the human patient, which showed the same overall response in PDX mice as in humans, however, more research is needed to understand how similar PDX tumors are to patient response when chemotherapy has been given.

SLAM microscopy has been used for some in vivo cell tracking with time-lapse imaging^22^, but remains underexplored in PDX models as well, and could provide further insight on cell migration and the cell death processes; future studies could also explore a window model for longitudinal time-lapse imaging of tumors during treatment. Interestingly, our results showed significant differences between in vivo and ex vivo images of the same region for a variety of image metrics (Fig. S1). Despite this, it is possible that ex vivo imaging could show the same trends in image features across groups and give rise to a similar interpretation of results. However, it has also been shown that nonlinear optical signatures from ex vivo tissue specimens can change over time on the timescale of minutes, even with tissue perfusion and culture conditions, so time may be a confounding factor in experiments using nonlinear optical microscopy to examine metabolic differences in ex vivo tissue specimens^23^. Further studies should also take advantage of the multi-modality of SLAM microscopy and apply this advantage for higher dimensional analysis such as principal component analysis, artificial intelligence, or deep learning, however, that was not appropriate for this dataset based on the small number of mice used and confounding interanimal variability. These computational methods could also be used to study if SLAM microscopy images of pretreatment or tumors in the early stages of treatment could be used to predict treatment responsiveness. Additional work could compare SLAM microscopy with other methods to assess chemotherapy response in vivo, such as FDG-PET, another metabolic imaging method, which was recently shown to predict the pathological treatment response of neoadjuvant chemotherapy in PDAC^43^.

There is a need for new approaches to study the PDAC microenvironment, specifically understanding the relationship between metabolism and treatment response to establish faster and more efficient personalized treatment to overcome chemoresistance^2,14^. In this study, optical signatures from SLAM microscopy were used to investigate the pharmacological response of responsive and resistant chemotherapy regimens in human PDAC in PDX mouse models. SLAM microscopy provides various structural and functional image metrics that are linked to the responsiveness of chemotherapy. Furthermore, tumors responsive and resistant to treatment can show a similar early metabolic surge, but the responses clearly diverge at 5 weeks of treatment. Resistant tumors show elevated levels of EVs and return to untreated control levels of tumor metabolism, whereas tumors responding to treatment continue to show elevated markers of cell death. These findings provide further evidence for responsive subpopulations of tumor cells within “resistant” tumors, and also further implicate EVs as playing a role in chemotherapy resistance. This feasibility study shows the promise of these label-free optical imaging metrics and signatures in the further investigation of chemotherapy response in the live tumor microenvironment.

## Methods

### PDX samples

All ethical regulations relevant to human research participants were followed. The collection of human tissue to create and confirm the PDX models was reviewed and approved by the Mayo Clinic Institutional Review Board (IRB) and the Institutional Animal Care and Use Committee (IACUC). Patients provided informed consent for their tumor tissue to be used for the IRB-approved protocols. The tissue samples were collected during surgery from pathologically confirmed pancreatic tumors. The development and validation process of the PDX models used has been described previously^44,45^.

PDX samples with known responsiveness or resistance to chemotherapy were used in this study. The human response was previously evaluated in patients undergoing neoadjuvant chemotherapy. To evaluate the human response, several parameters are considered including anatomic (such as cross-sectional imaging), biochemical (such as blood testing on CA 19-9), and metabolic (such as functional PET imaging if available) responses over time. After tumor excision, PDX samples were implanted in mice and treated with different or similar chemotherapy treatments to examine response (Table 3). In PDX mice, response is determined by changes in tumor volume during in vivo treatment. The first patient, whose tissue was used in group 1, underwent a treatment similar to FIRINOX and demonstrated a favorable response to their tumor. The second patient, whose tissue contributed to group 2, was subjected to Gem/NabP and exhibited resistance. The third patient, whose tissue was utilized in groups 3 and 4, received Gem/NabP and displayed responsiveness to the treatment. The study of PDX tumors in mice revealed that Group 1 responded well to FIRINOX, much like the patient did. Group 2 showed resistance to FIRINOX, which was not tested on the patient. The researchers created PDX mice for groups 3 and 4 using tumor tissue from the same patient, patient 3. PDX mice created using this tumor displayed resistance to FIRINOX, which was not tested on the patient, and responsiveness to Gem/NabP, consistent with patient response.

**Table 3:** Chemotherapy responses of tumors in human patients and PDX mice.

Patient #	Neoadjuvant therapy received by human patient	Response in human patient	Response in corresponding PDX mice
1	5-FU based regimen like FIRINOX	Responsive to 5-FU based regimen	Responsive to FIRINOX
2	Gem/NabP	Resistant to Gem/NabP	Resistant to FIRNOX (Not received by the patient)
3	Gem/NabP	Responsive to Gem/NabP	Resistant to FIRNOX (Not received by the patient) Responsive to Gem/NabP

Responsiveness or resistance to neoadjuvant chemotherapy received by human patients prior to tumor removal is indicated. After tumor was removed, the response of that tumor was recorded in PDX mice to either FIRINOX or Gem/NabP.

### PDX mice preparation

All animal experiments received ethical approval and were performed under protocols approved by the IACUC for Mayo Clinic and the University of Illinois Urbana-Champaign. We have complied with all relevant ethical regulations for animal testing. PDX mouse models were created using a previously established protocol^19^. To implant the PDX sample into mice, fragments (2 × 2 × 2 mm^3^) of human PDAC tissue from 3 different patients were surgically implanted inside a subcutaneous space superior to the left flank of 8-week-old female NOD SCID mice (NOD.CB17-Prkdcscid/NCrCrl. Strain code: 394. Charles River) at the Mayo Clinic (Rochester, MN). PDX mice were then shipped to the University of Illinois Urbana-Champaign (Urbana, IL) and housed in a separated sterile room under biosafety level 2 conditions. All treatments were administered in a biosafety cabinet. For this study, 20 mice were divided into 4 groups; mice in group 1 were implanted with tumor samples from patient 1 and received FIRINOX treatment (or saline for untreated controls), mice in group 2 were implanted with tumor samples from patient 2 and received FIRINOX treatment (or saline for untreated controls), mice in group 3 were implanted with tumor samples from patient 3 and received FIRINOX treatment (or saline for untreated controls), mice in group 4 were implanted with tumor samples from patient 3 and received Gem/NabP treatment (or saline for untreated controls) (Fig. 1). Each week, the tumor size (measured through the skin with a caliper) and the weights of the mice were recorded (Fig. S5). At each timepoint (group 1: 1 week, 5 weeks; group 2: 1 week, 3 weeks; groups 3 and 4: 1 week, 3 weeks, 5 weeks), one untreated control and one chemotherapy-treated mouse were imaged from each group.

### Chemotherapy treatment for PDX mice

Treatment started after the tumor of each mouse within the group reached a specified volume (Fig. 1). For the first group, treatment started when tumors became palpable (mean volume = 5 mm^3^). For the second group, treatment started after tumor reached a volume of 200 mm^3^. For the third and the fourth group, treatment started after tumor reached a volume of 100 mm^3^. Two different chemotherapy regimens were administered via intraperitoneal injections; untreated control mice received saline injections of matched volumes on the same treatment schedule. FIRINOX was a combination of 5-Fluorouracil (5-FU), Irinotecan (IRINO), and Oxaliplatin (OX) with a starting dose of 50 mg/kg of 5-FU, 15 mg/kg of IRINO, and 2 mg/kg of OX, and full dose of 75 mg/kg of 5-FU, 30 mg/kg of IRINO, and 4 mg/kg of OX. Treatment was administered three times a week with 5-FU on Mondays, OX on Wednesdays, and IRINO on Fridays. Gem/NabP was a combination of Gemcitabine (Gem) and nab-Paclitaxel (NabP, Abraxane) with a starting dose of 20 mg/kg of Gem and 5 mg/kg of NabP, and a full dose of 40 mg/kg Gem and 10 mg/mg NabP. Gem was administered once a week and NabP was administered daily. Starting doses were administered for the first week of treatment and full doses were administered for all following weeks.

### Surgical exposure for imaging of tumor tissue

Optical access to the tumor and its microenvironment was achieved by surgically creating a skin flap laterally over the tumor region. Mice were anesthetized with 2% isoflurane during surgical exposure and 1% isoflurane during in vivo imaging. After imaging, the animals were euthanized. The skin flap was dissected for ex vivo imaging for a few select specimens. After imaging, tissue was fixed for histopathology analysis.

### H&E staining and histology

After SLAM imaging, tumor samples were excised from the animals, fixed in 10% formalin, embedded in paraffin, and sectioned at 5 μm on a microtome (Leica). Slides were then stained with hematoxylin and eosin for microscopic observations. Briefly, microscope slides were deparaffinized in xylene through graded alcohols, stained with Harris’s hematoxylin (5 min) followed by Eosin (30 s) before dehydrating through graded alcohols into xylene prior to applying a coverslip. Microscope slides were digitized using a slide scanner (Nanozoomer 2.0 RV w/fluorescence, Hamamatsu). Tumor presence was confirmed by a board-certified pathologist.

### Microscope setup and imaging

A custom-built SLAM microscope (Fig. S6) was used for intravital imaging^22^. In brief, tailored 1110 ± 30 nm wavelength pulses at a repetition rate of 20 MHz were focused on the tissue sites by an objective lens (XLPLN25XWMP2, NA = 1.05 Olympus) with an average power of 14 mW on the sample. Optical filters were used to separate emitted signal into four channels: 365–375 nm (THG channel), 420–480 nm (3PF/NAD(P)H channel), 540–570 nm (SHG channel), and 580–640 nm (2PF/FAD channel). Channels were collected simultaneously by an array of four photomultiplier tubes (H7421-40, Hamamatsu). Following surgical exposure of the tumor tissue while under anesthesia, mice were placed on a 3-D piezoelectric stage for imaging. The number of fields of view acquired were determined based on the amount of visible tumor. Animals were monitored during imaging to ensure their breathing was present and constant; if the animal showed signs of instability the imaging session was ended. Along with approximate tumor size, this contributed to uneven numbers of fields of view collected for each mouse. After acquisition, certain fields of view were excluded from analysis due to large breathing artifacts. Multiple sub-images were captured for mosaic images ranging from 1 × 1 mm^2^ to 8.4 × 12.6 mm^2^.

### Image analysis

Sub-images for mosaic images were stitched with custom MATLAB (Mathworks, Natick, MA) script. Images were divided into 600 × 600 pixel (300 × 300 μm^2^) tiles for analysis; total number of tiles used is given in Table S1. Image metric calculations including mean channel intensities, ORR, fraction of NAD(P)H-rich pixels, collagen alignment ratio, and EV segmentation and analysis was performed with custom MATLAB scripts.

### Statistics and Reproducibility

Kruskal-Wallis one way ANOVAs were used to determine p-values for significant differences between variables between control and treated mice. Statistical comparisons for treated mice across different groups was not performed due to differences in control mice, likely due to differences in tumor size and origin (Fig. S2). A *p* value of < 0.05 was considered significant. One mouse was examined per timepoint per treatment due to the nature of this feasibility study. Many different unique fields of view were acquired for each mouse (Table S1) to account for intra-tumor heterogeneity. The small number of mice used in this feasibility study warrants larger-scale studies to determine the reproducibility of these results.

### Reporting summary

Further information on research design is available in the Nature Portfolio Reporting Summary linked to this article.

### Supplementary information

Supplemental Material

Reporting Summary

## Data Availability

The data from this study are publicly available via Open Science Framework (OSF) and are freely available to view and download: https://osf.io/gfmzk/?view_only=2d993bca1a5c49c8972d6706ee2895b5^46^.

## Code availability

The codes to perform the analyses are publicly available via Open Science Framework (OSF) and are freely available to view and download: https://osf.io/gfmzk/?view_only=2d993bca1a5c49c8972d6706ee2895b5^46^.
