# Supplementary material for: In vivo label-free optical signatures of chemotherapy response in human pancreatic ductal adenocarcinoma patient-derived xenografts
Source: Commun Biol. 2023 Sep 25;6:980. doi: 10.1038/s42003-023-05368-y (PMC10520051; doi:10.1038/s42003-023-05368-y)
Supplement: Supplementary file 1 — Supplemental Material [file 42003_2023_5368_MOESM1_ESM.docx]

Supplementary Materials for

***In vivo* label-free optical signatures of chemotherapy response in human pancreatic ductal adenocarcinoma patient-derived xenografts**

Jaena Park, Janet E. Sorrells, Eric J. Chaney, Amro M. Abdelrahman, Jennifer A. Yonkus, Jennifer Leiting, Heidi Nelson, Jonathan J. Harrington, Edita Aksamitiene, Marina Marjanovic, Peter D. Groves, Colleen Bushell, Mark J. Truty, Stephen A. Boppart*

*Corresponding author: [boppart@illinois.edu](mailto:boppart@illinois.edu)

**This PDF file includes:**

Figs. S1 to S6

Table S1


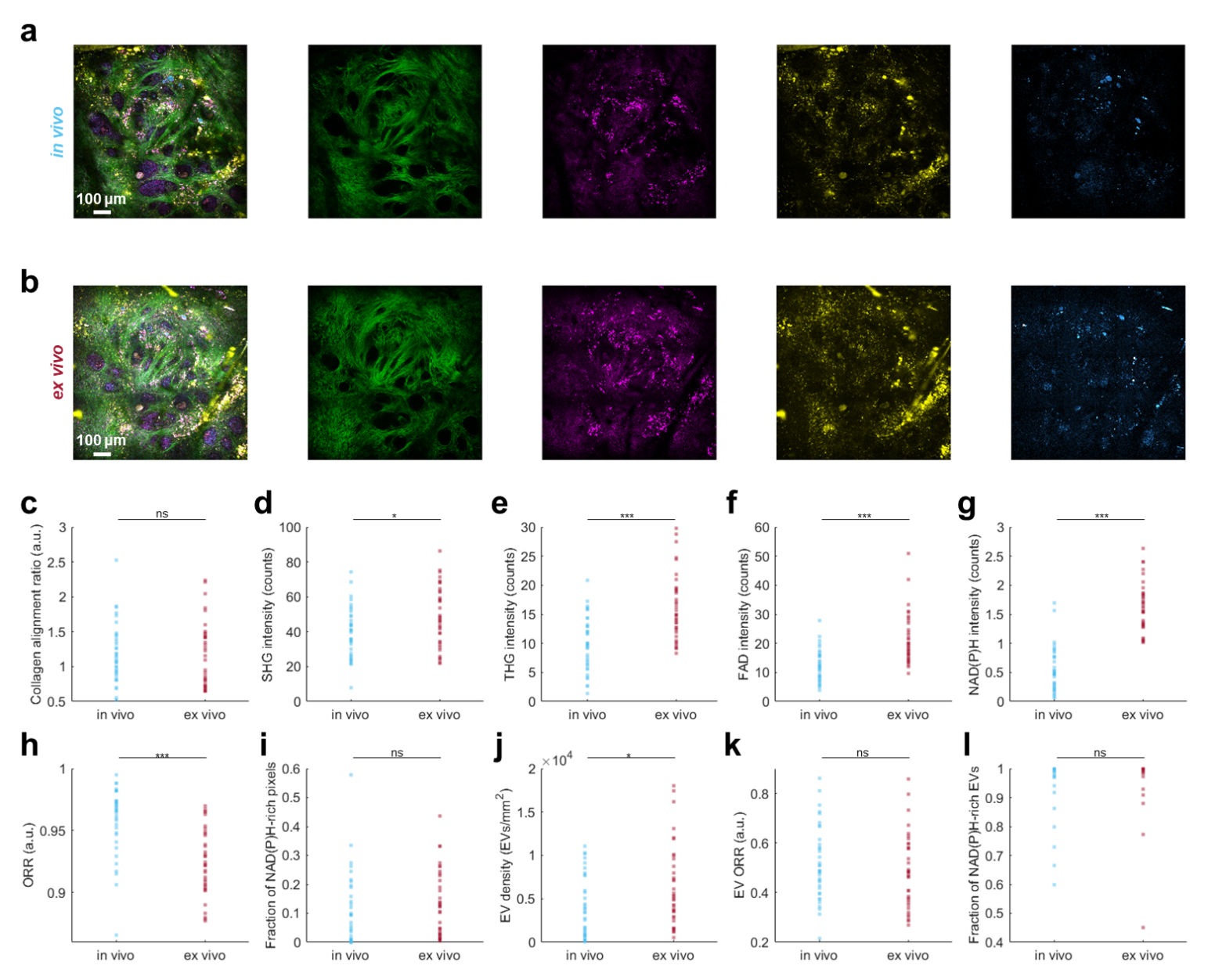


Fig. S1. Comparison of in vivo and ex vivo SLAM images and features. (a) Composite SLAM image (left), consisting of SHG, THG, 2PF, and 3PF channels for in vivo tumor. (b) Composite SLAM image (left), consisting of SHG, THG, 2PF, and 3PF channels for ex vivo tumor. Image features for in vivo (light blue) and ex vivo are provided for: (c) collagen alignment, (d) SHG intensity, (e) THG intensity, (f) FAD intensity, (g) NAD(P)H intensity, (h) tumor region ORR, (i) fraction of NAD(P)H-rich pixels, (j) EV density, (k) EV ORR, (l) fraction of NAD(P)H-rich EVs. Scale bar: 100 μm (same for all images). ns: not significant; *: p < 0.05; ** p < 0.01; *** p < 0.001.


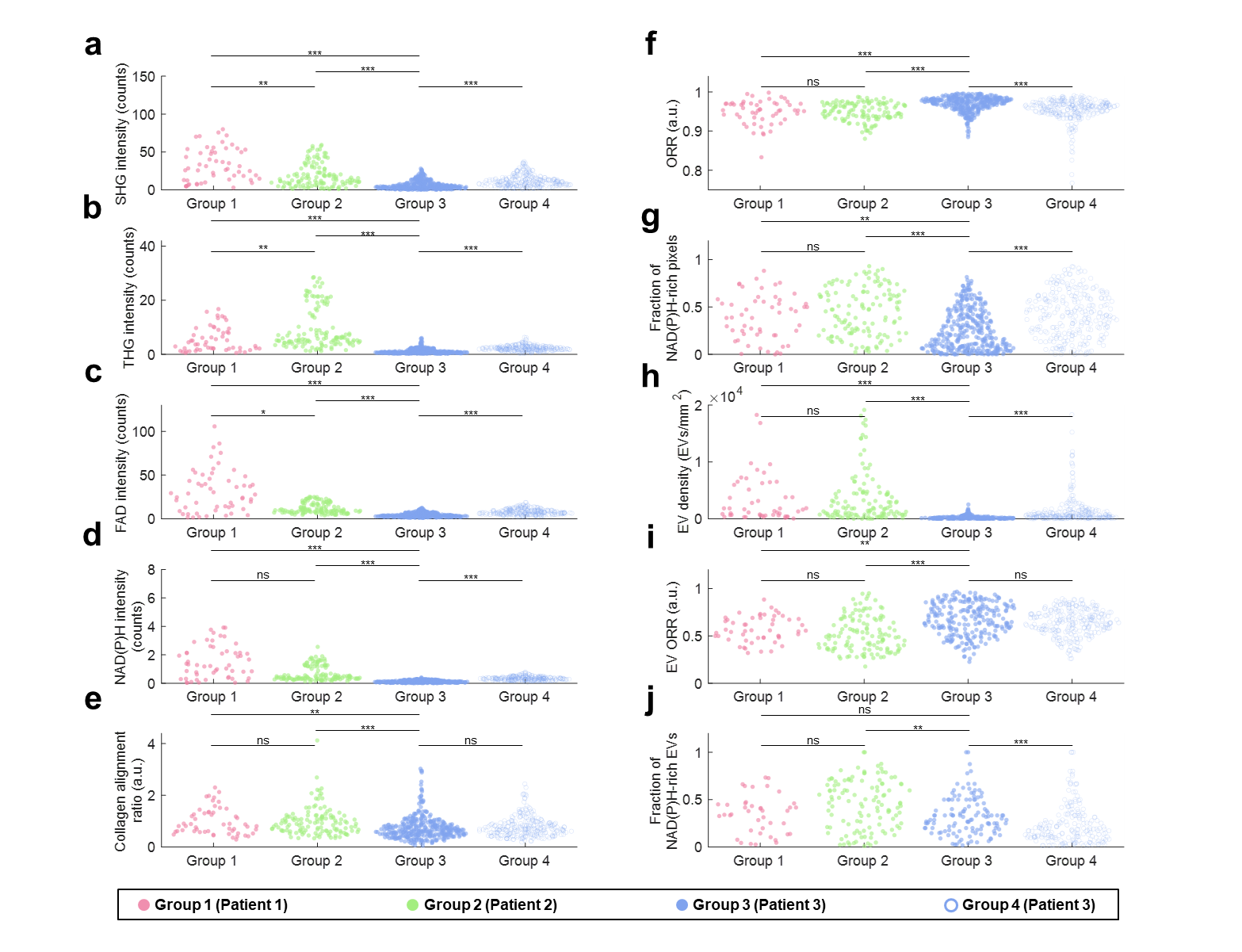


Fig. S2. SLAM image features for all untreated control tumors. All mosaic images were divided into tiles of 600×600 pixels (300×300 μm^2^), all timepoints were pooled together, and the following image metrics were computed for each tile: (a) SHG mean intensity in photon counts per pixel, (b) THG mean intensity in photon counts per pixel, (c) 2PF FAD mean intensity in photon counts per pixel, (d) 3PF NAD(P)H mean intensity in photon counts per pixel, (e) collagen alignment ratio computer from Fourier analysis of SHG channel, (f) mean optical redox ratio (ORR) of segmented tumor region within tile, (g) fraction of NAD(P)H-rich pixels of segmented tumor region within tile, defined as pixels with below-mean ORR, (h) segmented EV density in EVs/mm^2^, (i) mean EV ORR, (j) fraction of NAD(P)H-rich EVs, defined as the fraction of EVs within the tile with below-mean ORR. For groups 1 and 2, n = 2 mice; for groups 3 and 4, n = 3 mice. ns: not significant; *: p < 0.05; ** p < 0.01; *** p < 0.001.


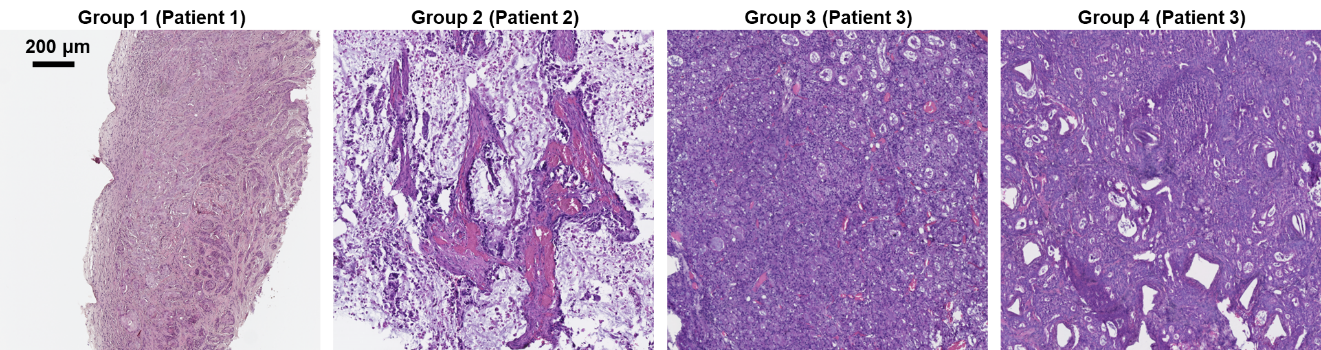


Fig. S3. Comparison of H&E-stained histology sections for untreated control tumors. Inter-tumor heterogeneity is visibly present among these samples. Scale bar: 200 μm (same for all images).


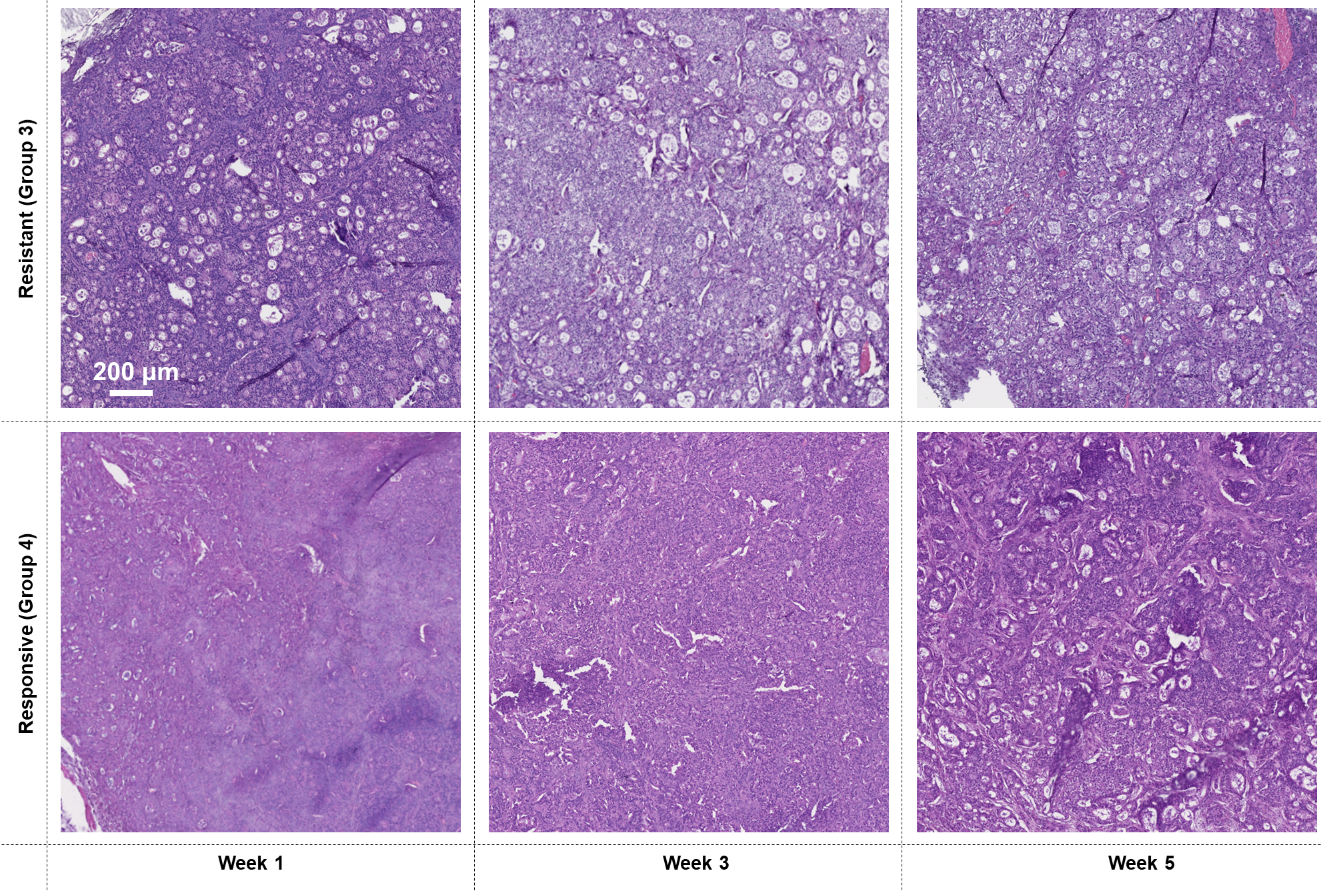


Fig. S4. Comparison of H&E-stained histology sections for groups 3 and 4 to visualize differences between tumors resistant and responsive to treatment. Scale bar: 200 μm (same for all images).


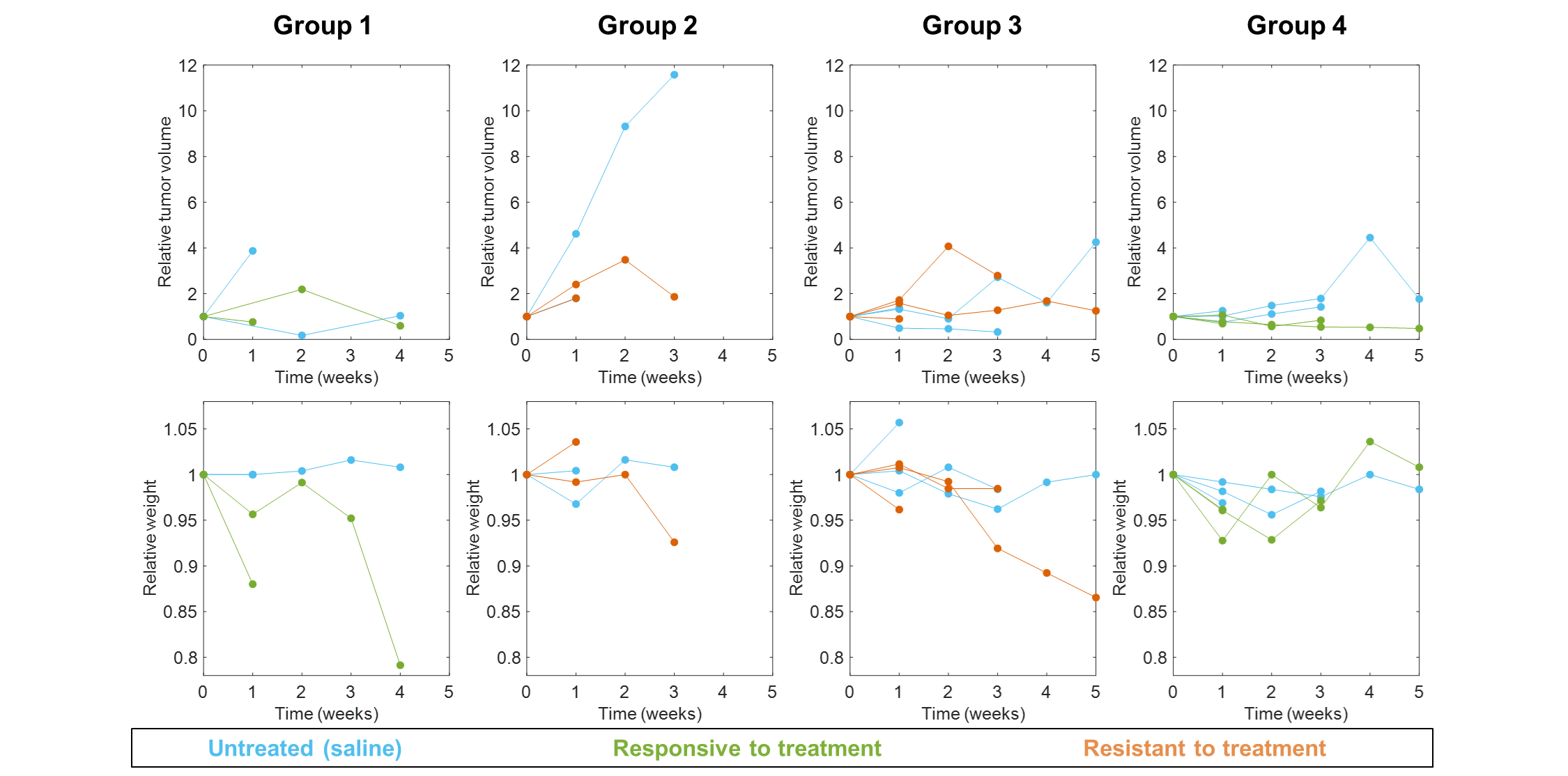


Fig. S5. Relative tumor volume and mouse weight normalized to the start of treatment. Each set of data points and corresponding line represents an individual mouse.


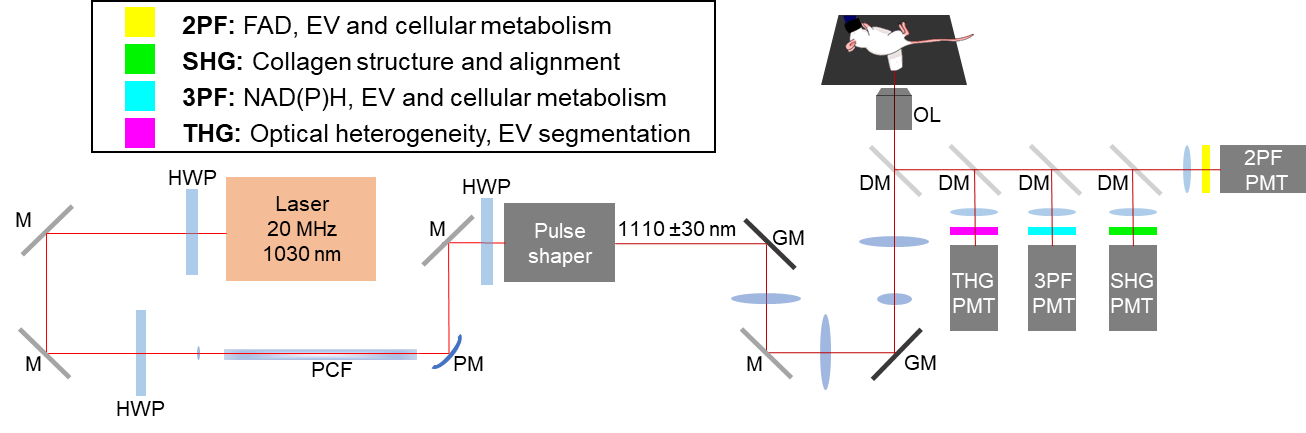


Fig. S6. Schematic of the SLAM microscope. HWP: half wave plate; M: mirror; PCF: photonic crystal fiber; PM: parabolic mirror; GM: galvo mirror; OL: objective lens; PMT: photomultiplier tube; DM: dichroic mirror; 2PF: two photon fluorescence; SHG: second harmonic generation; 3PF: three photon fluorescence; THG: third harmonic generation; FAD: flavin adenine dinucleotide; EV: extracellular vesicle; NAD(P)H: reduced nicotinamide adenine dinucleotide (phosphate).

Table S1. Number of tiles (600 × 600 square pixels, 300 × 300 μm^2^) for each group, treatment, and timepoint.

|  | **Week 1** | **Week 3** | **Week 5** |
| --- | --- | --- | --- |
| **Group 1, untreated control** | 33 | 0 | 8 |
| **Group 1, FIRINOX** | 54 | 0 | 18 |
| **Group 2, untreated control** | 9 | 106 | 0 |
| **Group 2, FIRINOX** | 9 | 9 | 0 |
| **Group 3, untreated control** | 98 | 6 | 108 |
| **Group 3, FIRINOX** | 43 | 107 | 99 |
| **Group 4, untreated control** | 8 | 34 | 81 |
| **Group 4, Gem/NabP** | 18 | 96 | 137 |
