# Supplementary material for: In vivo label-free optical signatures of chemotherapy response in human pancreatic ductal adenocarcinoma patient-derived xenografts
Source: Commun Biol. 2023 Sep 25;6:980. doi: 10.1038/s42003-023-05368-y (PMC10520051; doi:10.1038/s42003-023-05368-y)
Supplement: Supplementary file 2 — Reporting Summary [file 42003_2023_5368_MOESM2_ESM.pdf]

nature portfolio

Corresponding author(s): Janet Sorrells
Stephen A. Boppart

Last updated by author(s): 8/24/2023

Reporting Summary

Nature Portfolio wishes to improve the reproducibility of the work that we publish. This form provides structure for consistency and transparency in reporting. For further information on Nature Portfolio policies, see our Editorial Policies and the Editorial Policy Checklist.

Statistics

For all statistical analyses, confirm that the following items are present in the figure legend, table legend, main text, or Methods section.

n/a	Confirmed	
☐	☒	The exact sample size (*n*) for each experimental group/condition, given as a discrete number and unit of measurement
☐	☒	A statement on whether measurements were taken from distinct samples or whether the same sample was measured repeatedly
☐	☒	The statistical test(s) used AND whether they are one- or two-sided<br>*Only common tests should be described solely by name; describe more complex techniques in the Methods section.*
☐	☒	A description of all covariates tested
☐	☒	A description of any assumptions or corrections, such as tests of normality and adjustment for multiple comparisons
☐	☒	A full description of the statistical parameters including central tendency (e.g. means) or other basic estimates (e.g. regression coefficient) AND variation (e.g. standard deviation) or associated estimates of uncertainty (e.g. confidence intervals)
☐	☒	For null hypothesis testing, the test statistic (e.g. *F*, *t*, *r*) with confidence intervals, effect sizes, degrees of freedom and *P* value noted<br>*Give P values as exact values whenever suitable.*
☒	☐	For Bayesian analysis, information on the choice of priors and Markov chain Monte Carlo settings
☒	☐	For hierarchical and complex designs, identification of the appropriate level for tests and full reporting of outcomes
☒	☐	Estimates of effect sizes (e.g. Cohen's *d*, Pearson's *r*), indicating how they were calculated

Our web collection on statistics for biologists contains articles on many of the points above.

Software and code

Policy information about availability of computer code

Data collection	Labview 2019 was used for data collection.
Data analysis	Custom Matlab code was used to analyze the data, and will be made publicly available upon publication via Open Source Network.

For manuscripts utilizing custom algorithms or software that are central to the research but not yet described in published literature, software must be made available to editors and reviewers. We strongly encourage code deposition in a community repository (e.g. GitHub). See the Nature Portfolio guidelines for submitting code & software for further information.

Data

Policy information about availability of data

All manuscripts must include a data availability statement. This statement should provide the following information, where applicable:

- Accession codes, unique identifiers, or web links for publicly available datasets
- A description of any restrictions on data availability
- For clinical datasets or third party data, please ensure that the statement adheres to our policy

The data and codes to perform the analyses are publicly available via Open Science Framework (OSF) and are freely available to view and download: https://osf.io/gfmzk/?view_only=2d993bca1a5c49c8972d6706ee2895b5 (51).

Research involving human participants, their data, or biological material

Policy information about studies with human participants or human data. See also policy information about sex, gender (identity/presentation), and sexual orientation and race, ethnicity and racism.

Reporting on sex and gender	NA
Reporting on race, ethnicity, or other socially relevant groupings	NA
Population characteristics	NA
Recruitment	Patients provided informed consent for their tumor tissue to be used for the IRB-approved protocols. The tissue samples were collected during surgery from pathologically confirmed pancreatic tumors.
Ethics oversight	All ethical regulations relevant to human research participants were followed. The collection of human tissue to create and confirm the PDX models was reviewed and approved by the Mayo Clinic Institutional Review Board (IRB) and the Institutional Animal Care and Use Committee (IACUC).

Note that full information on the approval of the study protocol must also be provided in the manuscript.

Field-specific reporting

Please select the one below that is the best fit for your research. If you are not sure, read the appropriate sections before making your selection.

☒ Life sciences ☐ Behavioural & social sciences ☐ Ecological, evolutionary & environmental sciences

For a reference copy of the document with all sections, see nature.com/documents/nr-reporting-summary-flat.pdf

Life sciences study design

All studies must disclose on these points even when the disclosure is negative.

Sample size	Sample size was chosen in order to carry out a small-scale feasibility study, based on tumor xenograft and mouse availability.
Data exclusions	Some mice were excluded from the study due to premature death.
Replication	Due to limitations on budget and xenograft availability, results were not replicated.
Randomization	Allocation was random.
Blinding	Investigators were not blinded due to the small number of mice and that team members were involved in both animal treatment and imaging.

Reporting for specific materials, systems and methods

We require information from authors about some types of materials, experimental systems and methods used in many studies. Here, indicate whether each material, system or method listed is relevant to your study. If you are not sure if a list item applies to your research, read the appropriate section before selecting a response.

Materials & experimental systems

n/a	Involved in the study	
☒	☐	Antibodies
☒	☐	Eukaryotic cell lines
☒	☐	Palaeontology and archaeology
☐	☒	Animals and other organisms
☒	☐	Clinical data
☒	☐	Dual use research of concern
☒	☐	Plants

Methods

n/a	Involved in the study	
☒	☐	ChIP-seq
☒	☐	Flow cytometry
☒	☐	MRI-based neuroimaging

Animals and other research organisms

Policy information about studies involving animals; ARRIVE guidelines recommended for reporting animal research, and Sex and Gender in Research

Laboratory animals	8-week-old female NOD SCID mice (NOD.CB17-Prkdcscid/NCrCrl. Strain code: 394. Charles River)
Wild animals	NA
Reporting on sex	Only female mice were used in order to reduce variability.
Field-collected samples	NA
Ethics oversight	All animal experiments received ethical approval and were performed under protocols approved by the IACUC for Mayo Clinic and the University of Illinois Urbana-Champaign.

Note that full information on the approval of the study protocol must also be provided in the manuscript.
